# Further insights into the thermodynamics of linear carbon chains for temperatures ranging from 13 to 300 K

**DOI:** 10.3762/bjnano.16.125

**Published:** 2025-10-20

**Authors:** Alexandre Rocha Paschoal, Thiago Alves de Moura, Juan S Rodríguez-Hernández, Carlos William de Araujo Paschoal, Yoong Ahm Kim, Morinobu Endo, Paulo T Araujo

**Affiliations:** 1 Department of Physics, Federal University of Ceara, 60440-900 Fortaleza, Ceara, Brazil; 2 Department of Physics and Astronomy, University of Alabama, Tuscaloosa, Alabama 35487, USA; 3 Departamento de Ensino, Instituto Federal de Educação, Ciência e Tecnologia do Ceará (IFCE), 62580-000, Acarau, Ceara, Brazil; 4 Department of Polymer Engineering, School of Polymer Science and Engineering, and Alan G. MacDiarmid Energy Research Institute, Chonnam National University, 77 Yongbong-ro, Buk-gu, Gwangju 61186, Republic of Korea; 5 Faculty of Engineering, Shinshu University, 4-17-1 Wakasato, Nagano-shi 380-8553, Japan

**Keywords:** carbon nanotubes, Debye model, Grüneisen parameter, linear carbon chains, Raman spectroscopy

## Abstract

It was recently shown that small bundles of linear carbon chains (LCC) encapsulated by double- and multi-wall carbon nanotubes (LCC@DWCNT and LCC@MWCNT, respectively) behave as Debye’s materials for temperatures as high as 293 K with an estimate that such materials could still withstand such characteristics for even higher temperatures (≈700 K). Using the Debye model, thermodynamic observables (internal energy, coefficient of linear thermal expansion, specific heat, thermal strain, and Grüneisen parameter at constant pressure) were empirically determined for the first time in the range of temperatures 70 < *T* < 293 K. These observables were all correlated with the C-band frequency (ω_LCC_) dependence on the temperature (*T*) and its first and second derivatives with relation to T, dω_LCC_/d*T*, and d^2^ω_LCC_/d*T*^2^. The C-band is a Raman spectroscopic signature for LCC, which is not only temperature-dependent but also dependent on the number of carbon atoms (*N*) constituting the LCC. In this present study, we extend these findings to temperatures ranging from 13 < *T* < 293 K, which provide more accurate values for both dω_LCC_/d*T* and d^2^ω_LCC_/d*T*^2^. The corrected values of these derivatives affect the Grüneisen parameters associated with the LCC, even though the other associated thermodynamic parameters remain essentially unchanged. Our measurements were performed in both isolated and small bundles of LCC@MWCNT, which allowed us to demonstrate that small bundles or isolated environments do not seem to influence the vibrational and thermodynamic properties measured.

## Introduction

Phonons, their mutual interactions (ph–ph interactions), and their interactions with electrons (e–ph interactions) play fundamental roles in how materials respond to electric (e.g., difference of potentials), thermal (e.g., temperature gradients), and mechanical (e.g., pressure variations) stimuli [1–23]. These responses are directly connected with electronic and transport properties, which in turn depend on the equilibrium between emission and absorption of phonons, and gain and loss of energy of carriers [1–2,10,17,24–28]. The phonon lifetime as well as the selection rules behind ph–ph and e–ph interactions determine the efficiency of such phonon emission and absorption [1–2,10,17,24–28]. Phonons need to be in an excited state to be emitted or absorbed. Once they decay to their ground state, they become unavailable. This decay process is often accomplished via three-phonon processes (called the Klemens’ channel) and via four-phonon processes [1–3,6–7,12–13,16,24]. It is widely known that pressure (*P*)- and temperature (*T*)-dependent phenomena are ruled by anharmonic ph–ph interactions, which are also driven by three- and four-phonon processes, and by e–ph interactions [1–29].

Therefore, phonon assignments in materials as well as the understanding of how such phonons relate to thermal and mechanical properties of the materials become of fundamental importance [1–30]. One important point to keep in mind is that ph–ph and e–ph interactions are also very susceptible to the dimensionality of the materials, and for one-dimensional (1D) materials, the selection rules behind such interactions are rather restricted [13,31–33]. These interactions are all quantum-related phenomena, and their ineffectiveness allows thermal and mechanical properties of materials to be described by semi-classical theories (such as the Debye’s theory that describes the behavior of materials with *T*), and their phonon frequencies might be directly connected with relevant parameters such as the Young’s modulus, the Grüneisen parameter, and thermal expansion coefficient [29–30]. This is the case with linear atomic chains constituted of carbon atoms [29–30].

Linear carbon chains (LCC) are 1D systems that are classified into two categories: polyynes (displaying alternating triple and single bonds between constituent carbon atoms) and cumulenes (displaying only double bonds between constituent carbon atoms) [31,34–40]. Cumulenes are metallic systems that, due to Peierls transition, are more unstable than polyynes, which present insulating properties with bandgaps whose sizes are dependent on the number of carbon atoms (*N*) constituting the chains [31,34–40]. The unique properties associated with LCC have attracted a great deal of attention in the scientific community. They are structures that present unique anharmonic behaviors [29–30,41–42], and they are claimed to possess one of the largest mechanical resistances among materials (including other carbon allotropic versions like graphene or nanotubes) [29,43–49], in addition to presenting unique conductive properties that place them ahead as ideal candidates for future developments in nanoelectronics [43–49]. Moreover, due to its simplicity, LCC are like textbook problems in which many simple, but powerful, theories can be tested [29–31].

Until recently, many challenging questions regarding LCC stability have been raised [39,50–53]. Most of these questions regard the stability of host-free LCC, and they are readily circumvented when the LCC are hosted by carbon nanotubes (CNT), when they are decorated with terminal groups such as the tris(3,5-di-*t*-butylphenyl)methyl, or when they are in colloidal environments [32,34,36–38,43,45,49–55]. Recently, single-wall (SW), double-wall (DW), and multiwall (MW) CNT have been used and considered ideal environments for fabricating stable LCC with up to 6000 carbon atoms [34,36–38,43,45,49–55]. Due to their 1D character, LCC are very simple structures, presenting rather simple electronic and phonon structures that are dependent on *N* [31–32]. When they are host-free, their phonon structures present longitudinal and transversal modes but their encapsulation by CNT inhibits transversal modes [29–32,55–59]. This inhibition seems to be confirmed in a recent work by Moura et al. [60], in which a novel Raman active longitudinal mode was observed for LCC, but no active transversal modes were observed despite theoretical predictions that suggested their existence.

The literature reports that LCC phonons possess long mean free paths (≈0.5–2.5 μm) and lifetimes (≈30–110 ps), which tend to make ph–ph interactions inefficient [31–32]. These mean free paths and lifetimes are considerably larger when compared with other carbon materials [13,31–33]. In addition, several other works [29–32,55–59] have pointed out that CNT provide conditions that are sufficient to stabilize the LCC and inhibit transversal vibrations, while keeping CNT and LCC properties disentangled. In fact, this remains true even when the LCC@CNT systems are submitted to high pressures [29,55–57]. The literature has also shown that many chain-like quasi-1D materials constituted of C_60_ bulky-balls submitted to various conditions of pressure and temperature remain harmonic with properties that are independent of the properties from their hosting CNT [61–65]. This suggests that 1D-like materials also present inefficient ph–ph interactions, and that mutual interactions between chain-like structures and their hosts are second-order effects [61–65]. Sulfur chains inside SWCNT have also demonstrated enhanced field-emission properties and outstanding gas-sensing properties [66–67], which once again corroborate the idea that the hosting CNT primarily serve as a stabilizing environment rather than one that alters the properties of the materials.

In this context, Costa and collaborators [30], demonstrated that LCC encapsulated by both multi-wall (LCC@MWCNT) and double-wall (LCC@DWCNT) carbon nanotubes are materials whose thermal properties can be described by the Debye model [30]. The reason is that the responses of materials to changing temperatures usually come from two contributions: (1) the lattice thermal expansion (LTE), associated with e–ph interactions; and (2) anharmonic effects, associated with ph–ph interactions. As is widely known, the Debye model does not consider ph–ph interactions but do describe quite well contributions associated with LTE [1,30]. Their work [30] was the first in the literature to provide experimental values for LCC internal energy per *N* (u), specific heat (*c*_v_), coefficient of linear thermal expansion (α), thermal strain (ε_T_), and Grüneisen parameter at constant pressure (γ_p_).

The present work is intended to explore some important points that were left open by Costa and collaborators [30]. These points mainly regard the influence of the number of CNT walls in the LCC thermodynamic parameters as well as the responses of the system when measured isolated (or in very small bundles) and when measured in bundles. In addition, the current work extends the study to even lower temperatures (i.e., 13 K) when compared with previous data, whose minimum temperature stood around 70 K. Our analysis follows the same protocols described in reference [30]: the temperature evolution of the longitudinal optical phonon (so-called C-band), which is Raman active with frequencies (ω_LCC_) around 1850 cm^−1^, is thoroughly tracked and ω_LCC_ is used to indirectly access important thermodynamic parameters associated with LCC. Note that the C-band is a spectroscopic signature widely used to identify distinct LCC since ω_LCC_ is dependent on the number of carbon atoms forming the chain (i.e., ω_LCC_ is size/length dependent).

## Results and Discussion

The LCC encapsulated by multiwalled CNT (LCC@MWCNT) were synthesized using arc discharge [53]. The purity of MWCNT regarding nanoparticles is ≈80% with average diameters of 10.4 nm (average length of 2.3 mm). The LCC@CNT filling ratio is ≈80% [53]. The samples were dispersed in acetone and sonicated for 2 h and then drop casted onto a Si wafer of ≈1 cm^2^ area. Raman spectra were acquired with a 20× objective lens in a backscattering geometry using Jobin Yvon Horiba T64000 spectrometer (1800 lines/mm grating). Samples were resonantly excited with 514.5 nm (2.41 eV) and 568.2 nm (2.18 eV) (Coherent Innova 70C Ar and Kr ion lasers).

**Figure 1:**
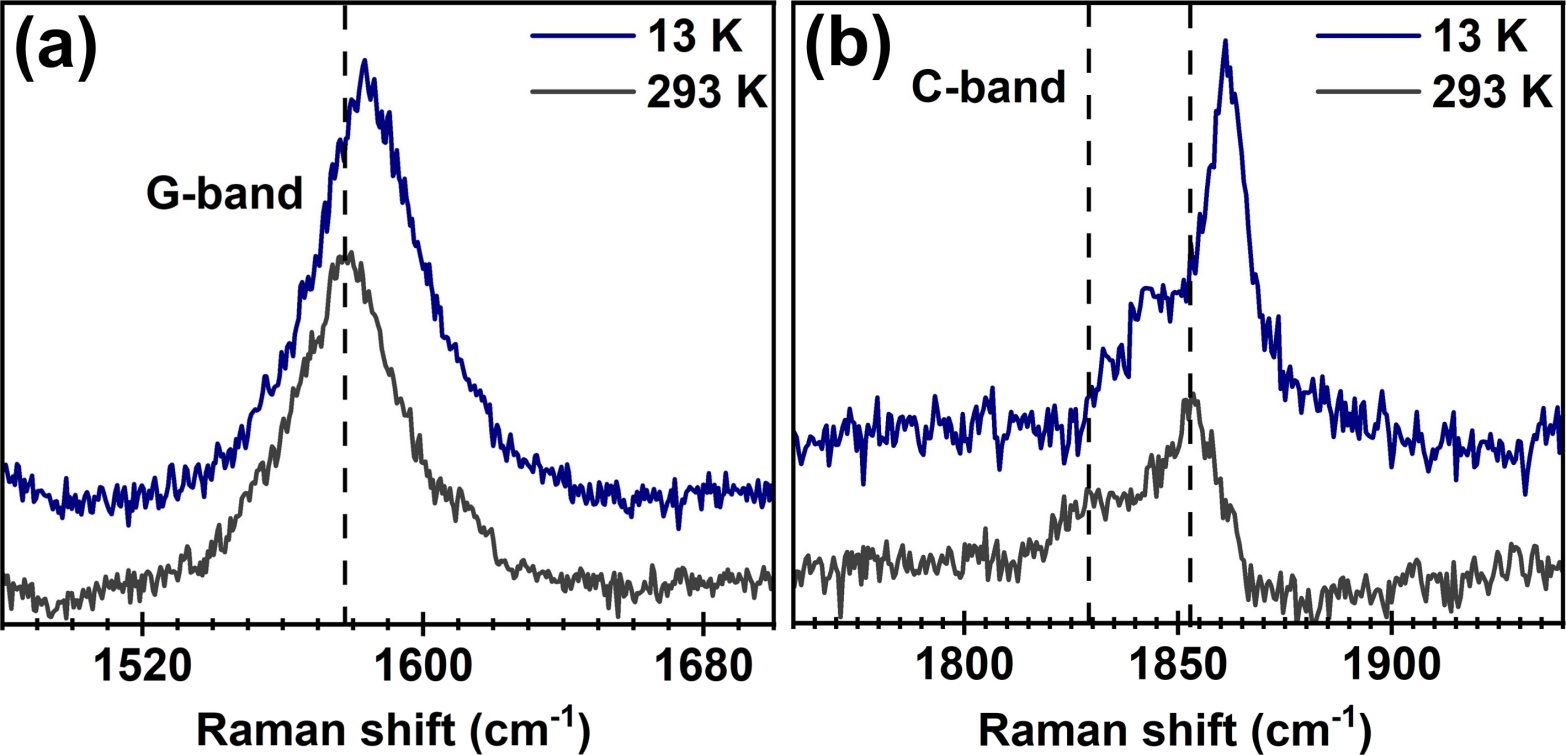
(a) and (b) show representative Raman spectra acquired from LCC@MWCNT at 13 K (solid navy blue curves) and 193 K (solid dark gray curves). The G-bands in (a) are associated with MWCNT, while the C-bands in (b) are associated with LCC. The vertical dashed lines are guides for the eyes. Supporting Information File 1, Figure S1 brings additional representative spectra.

**Figure 2:**
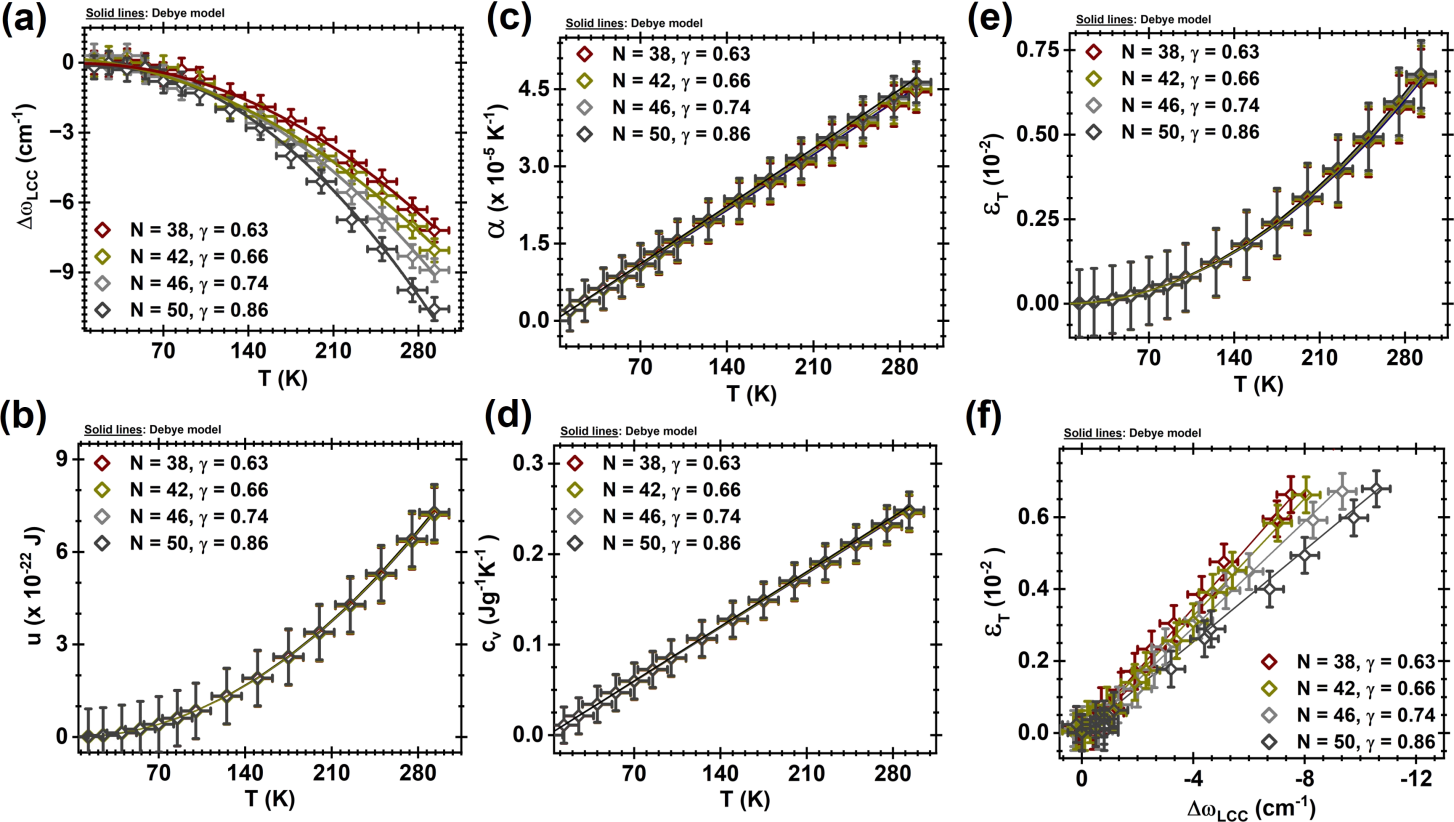
(a) Experimental Δω_LCC_(*T*) evolution with *T*; (b) the energy per *N*, u(*T*), presents a quadratic, universal, and unified behavior with *T*; (c) α(*T*) shows a linear universal behavior with *T*; (d) the heat capacity per *N*, *c*_V_(*T*), presents a linear, universal and unified behavior with *T*; (e) A *T*^2^ universal dependence is observed for the thermal strain ε_T_(*T*); (f) Every LCC presents a distinct linear dependence of ε_T_ with Δω_LCC_(*T*). Note that four representative LCC are shown here. The full set of LCC is presented in Figure S2 in Supporting Information File 1.

The Raman spectra of LCC@MWCNT were acquired at low temperatures ranging from 13 to 293 K. Figure 1 shows representative spectra at 13 K and at 293 K, where temperature-dependent frequency shifts are clearly observed for both G- and C-bands (G-band comes from MWCNT). The LCC C-band was fitted with four Lorentzian curves for the spectra collected with the 514.5 nm (2.41 eV) excitation source, and two Lorentzian curves for the spectra collected with the 568.2 nm (2.18 eV) excitation source. Each Lorentzian represents a distinct LCC. Representative Raman spectra and respective fittings are shown in Supporting Information File 1, Figure S1. In Figure 2, *N* was estimated considering that ω_LCC_ is proportional to *N*^−1^ [29,51]. It is important to recall that the association between *N* and ω_LCC_ is only a reliable approximation. This number *N* is used here to correlate the length of LCC with the thermodynamic variables studied, and such approximation does not impair the analysis and conclusions of this paper. Finally, for the sake of clarity, Figure 2 shows results for representative LCC, while the full set of LCC is shown in Supporting Information File 1, Figure S2.

In total, four LCC with *N* = 38, 42, 46, and 50 (γ_P_ = 0.63, 0.66, 0.74, and 0.86) were identified using 514.5 nm, while two LCC with *N* = 40 and 50 (γ_P_ = 0.67 and 0.81, respectively) were identified using 568.2 nm. In agreement with the literature [29–30], the use of different laser lines does not influence the response of the LCC@MWCNT to different *T*, but it might excite LCC with distinct *N* (LCC have their bandgap proportional to *N*^−1^; the smaller the chain, the larger the bandgap). Figure 2 corroborates this claim: ω_LCC_ for similar LCC possess similar dependence on *T*. As previously discussed, ω_LCC_ associated with each identified LCC is used as a probe to obtain the following thermodynamic properties as a function of *T* (*T* ranging from 13 to 293 K): *u*(*T*), α(*T*), *c*_v_(*T*), ε_T_(*T*), and γ_P_(*T*).

**Figure 3:**
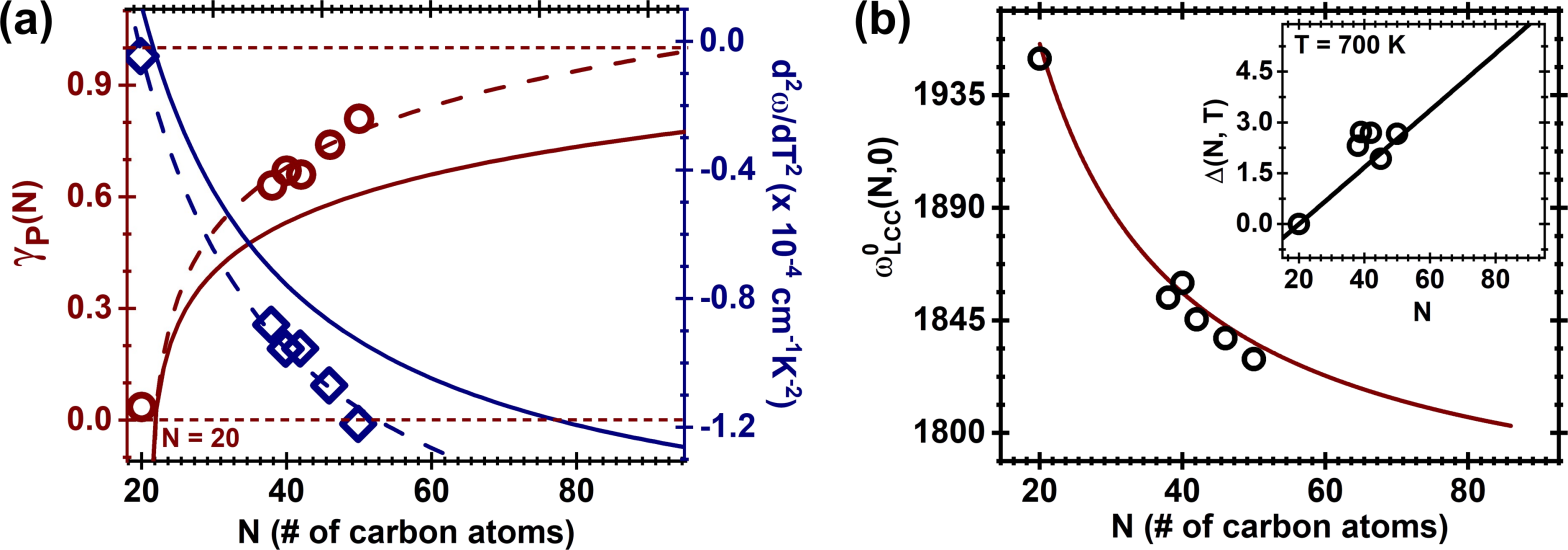
(a) Universal dependences with *N* for both γ_P_(*N*) = ln(*N* − 20)^0.23^ (burgundy solid lines) and 
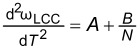
 (navy blue solid line with *A* = 1.88 × 10^−4^ cm^−1^·K^−2^ and *B* = 3.7 × 10^−3^ cm^−1^·K^−2^). The symbols (burgundy circles and navy blue diamonds for γ_P_ and 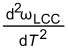, respectively) represent the experimental data. (b) 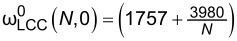 cm^−1^, where 
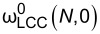
 is the C-band frequency at *T* = 0 K. The open circles represent the experimental data. The inset shows a representative case at *T* = 700 K for 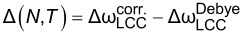, as discussed in the text and in [30].

The equations that correlate ω_LCC_ with these thermodynamic parameters are reported by Costa et al. [30] and reproduced in Supporting Information File 1 for reference (Equations S1–S6). Figure 2 shows a clear dependence of these properties with *N* and *T*, in accordance with previous work [30]. Costa and collaborators [30] studied these nanostructures under temperatures ranging from 70 to 293 K, but this work extends their results to temperatures as low as 13 K. Here, we measured isolated LCC@MWCNT under 568.2 nm (2.18 eV) excitation, while those acquired under 514.5 nm (2.41 eV) excitation was in very small bundles. For reference, Costa et al. [30] measured small bundles in their work. The plots shown in Figure 2 provide an answer to one of the questions we sought to address in this work: do small bundles or isolated environments influence the vibrational and thermodynamic properties measured? Figure 2 suggests that the answer is no. In fact, it is evident that the data obtained in both scenarios are similar within the error margin expected in these experiments. In addition, the Raman spectra as well as the independent evolution as a function of temperature of the Raman bands from CNT and LCC (see Figure 1 and Figure S1 in Supporting Information File 1) suggest that the interaction between distinct LCC, and LCC and CNT are not strong enough to affect their electronic and phonon structures. Therefore, in agreement with the literature [29–30], mutual interactions between LCC and CNT are second-order effects [29–30]. Moreover, if ph–ph interactions can be neglected, the ω_LCC_ variation with *T*(Δω_LCC_) should be well described by:


[1]

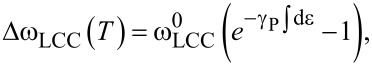



where 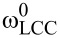 is the C-band frequency at *T* = 0 K, dε = α(*T*)d*T* is the thermal strain between *T* and *T* + d*T*, and γ_P_ is the *T*-independent Grüneisen parameter at constant *P*. Equation 1 in turn is expected to follow the empirical relation:


[2] $$\Delta \omega_{\text{LCC}}=\Delta \omega_{\text{LCC}}\left(T\right)-\omega_{\text{LCC}}^{0}=-\left(\frac{{\text{d}}^{2}\omega_{\text{LCC}}}{\text{d}T^{2}}\right)T^{2},$$

where the second derivative 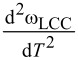 magnitude is *N*-dependent (see Figure 3). Figure 2a confirms that this is the case: the solid lines are fitting results using Equation 2. We are then well positioned to proceed with our analysis using the Debye model, whose associated equations (see Supporting Information File 1) depend on ω_LCC_, 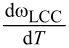, and 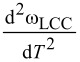 [30].

In one hand, Figure 2b shows that *u*(*T*) displays a *T*^2^ behavior; the values of *u*(*T*) at 13 K, 70 K, and 293 K are 1.42 × 10^−24^ J, 4.16 × 10^−23^ J, and 7.27× 10^−22^ J, respectively. On the other hand, α(*T*) and *c*_v_(*T*) (Figure 2c and 2d, respectively) vary linearly with *T* and do not show relevant dependence on *N*. Note that α(*T*) ranges from 4.40 × 10^−5^ K^−1^ (293 K) to 1.98 × 10^−6^ K^−1^ (13 K) with 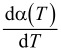 = 1.54 × 10^−7^ K^−2^, and *c*_v_(*T*) ranges from 0.25 J·g^−1^·K^−1^ (293 K) to 0.01 J·g^−1^·K^−1^ (13 K) with 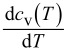 = 8.84 × 10^−4^ J·g^−1^·K^−2^; both are in good agreement with the literature [30,37,68]. The heat extracted from the LCC leads their shrinkage, generating an internal pressure that is associated with the thermal strain (ε_T_), which similarly with *u*(*T*), displays a *T*^2^ behavior (Figure 2e). This means that strains at 13 K (1.29 × 10^−7^, this work) are very similar to those at 70 K (3.74 × 10^−6^), while ε_T_ = 0.01 × 10^−2^ at ambient conditions. As discussed in the literature [29–30,51], in addition to depending on the temperature, ω_LCC_ is dependent on the chain length (i.e., *N*-dependent) as well (see Figure 3b). This is an expected behavior since the size of the chain affects the bond length alternation (BLA) strength of the polyynes. Figure 2a shows the evolution of ω_LCC_ with *T*, and again, it is noteworthy that no matter the length of the chain, there is a convergence of the data as the temperature decreases. Consequently, ε_T_ as a function Δω_LCC_ (see Figure 2f) also converges when ω_LCC_ → 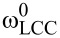. Note that in both cases, once again, the experimental data follow the Debye model up to 300 K. According to the data and the predictions above, Δω_LCC_ → 0 and ε_T_ → 0 when *T* → 0.

At this point, we are in good position to use Equation 1 to obtain γ_P_ associated with each measured chain, which in turn will deliver the γ_P_ dependence with *N* (Figure 3a). Note that once α(*T*) is known, γ_P_ becomes the only adjustable parameter in the equation. As anticipated earlier in the text, the found values for γ_P_ are in accordance with those reported in previous works [29–30], endorsing inefficient phonon–phonon coupling in this system. The open symbols shown in Figure 3a display 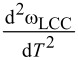 and γ_P_ as a function of *N*; both of them displaying an universal dependence on *N* given, respectively, by γ_P_(*N*) = ln(*N* − 20)^0.23^ and 
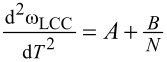
, where *A* = 1.88 × 10^−4^ cm^−1^·K^−2^ and *B* = 3.7 × 10^−3^ cm^−1^·K^−2^ (dashed lines in Figure 3a). The solid lines in Figure 3a represent γ_P_(*N*) and 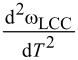
(*N*) found by Costa et al. [30] for temperatures as low as 70 K. It is noticeable that there is a slight discrepancy between the data found in this work and those published by Costa and collaborators [30], which is explained as follows: as discussed, γ_P_ and 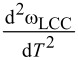 are calculated from the fitting of the experimental data from Figure 1a, and they are, therefore, heavily dependent on ω_LCC_(*T*,*N*) as a function of *T*, whose overall behavior is dependent on the range of temperatures used in the experiment. In the work by Costa et al. [30], the *T* range considered is limited to 70 < *T* < 300 K, while this paper extends it to 13 < *T* < 300 K. This newer range provides a more accurate prediction of 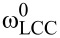 (i.e., ω_LCC_(*T*) for *T* = 0 K), and therefore, the extra data we bring in this paper provides a better low-temperature convergent behavior of ω_LCC_(*N*,*T*) and a better estimate for both 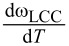 and 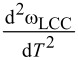, when compared with reference [30]. Since γ_P_ depends on Δω_LCC_(*T*), it is important to remind that, although the Debye model is understood to be valid even for temperatures beyond 300 K, the values we provide here are accurate up to 300 K only; these values of γ_P_ and 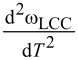 might always be updated with a broader range of temperatures.

Finally, Kastner et al. [51] have predicted 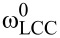 to follow 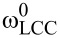
(*N*,0) = 1757 + 3890/*N* (in cm^−1^), which is plotted in the solid line of Figure 3b. The open circles represent our 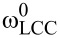 values extrapolated from the experimental data, which is in good agreement with Kastner and collaborators. Also, the Debye model formalism works very well for temperatures as high as 300 K and corrections are predicted for 300 < *T* < 700 K [30]. These corrections, however, continue to be small, as seen in the inset of Figure 3b, which plots the difference 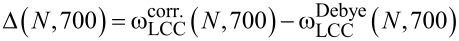 for the representative case at 700 K. It is worth reminding that, as seen in Equations S1 to S6 in Supporting Information File 1, the superscript “Debye” stands for the predicted values without any corrections involving 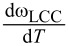 and 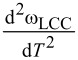, while the superscript “corr.” stands for values that are corrected by such derivatives. The solid line represents the same correction as predicted by Costa and collaborators [30]. The dispersion of the data (open circles) with relation to the black solid curve has the same origins as those associated with the values of γ_P_ and 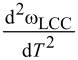.

## Conclusion

In summary, this paper investigates the thermodynamic properties of isolated and small bundles of LCC@MWCNT via Raman spectroscopy by tracking the C-band frequencies ω_LCC_ of LCC in the range of temperatures of 13 < *T* < 300 K. These range of temperatures provides more accurate values of 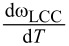 and 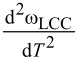, enhancing the reliability of the thermodynamic observables (*u*(*T*), α(*T*), *c*_v_(*T*), ε_T_(*T*), and γ_P_(*T*)). In addition, the data presented here further confirms that LCC may be well modelled using the Debye formalism even at ambient conditions. The thermodynamic observables indeed follow *N*-dependent universal laws with *T*. In this semiempirical model, the calculation of γ_P_ depends on the range of temperatures measured. The broader range of temperatures that this work considered allowed the authors to bring newly updated values of γ_P_. These values might, however, undergo further corrections when more experimental data is available for temperatures beyond 300 K. This work also confirms that equivalent thermodynamic properties are observed for small bundles and isolates LCC@MWCNT.

## Supporting Information

File 1 Additional figures and calculations.

## Data Availability

Additional research data generated and analyzed during this study is not shared.
