# Supplementary material for: Further insights into the thermodynamics of linear carbon chains for temperatures ranging from 13 to 300 K
Source: Beilstein J Nanotechnol. 2025 Oct 20;16:1818–25. doi: 10.3762/bjnano.16.125 (PMC12557416; doi:10.3762/bjnano.16.125)
Supplement: File 1 — Additional figures and calculations. [file Beilstein_J_Nanotechnol-16-1818-s001.pdf]

BEILSTEIN JOURNAL OF NANOTECHNOLOGY

Supporting Information

for

Further insights into the thermodynamics of linear carbon chains for temperatures ranging from 13 to 300 K

Alexandre Rocha Paschoal, Thiago Alves de Moura, Juan S. Rodríguez-Hernández, Carlos William de Araujo Paschoal, Yoong Ahm Kim, Morinobu Endo and Paulo T. Araujo

Beilstein J. Nanotechnol. **2025,** *16,* 1818–1825. doi:10.3762/bjnano.16.125

Additional figures and calculations

License and Terms: This is a supporting information file under the terms of the Creative Commons Attribution License (https://creativecommons.org/licenses/by/4.0). Please note that the reuse, redistribution and reproduction in particular requires that the author(s) and source are credited and that individual graphics may be subject to special legal provisions.
The license is subject to the *Beilstein Journal of Nanotechnology* terms and conditions: (https://www.beilstein-journals.org/bjnano/terms)

Representative Raman spectra:

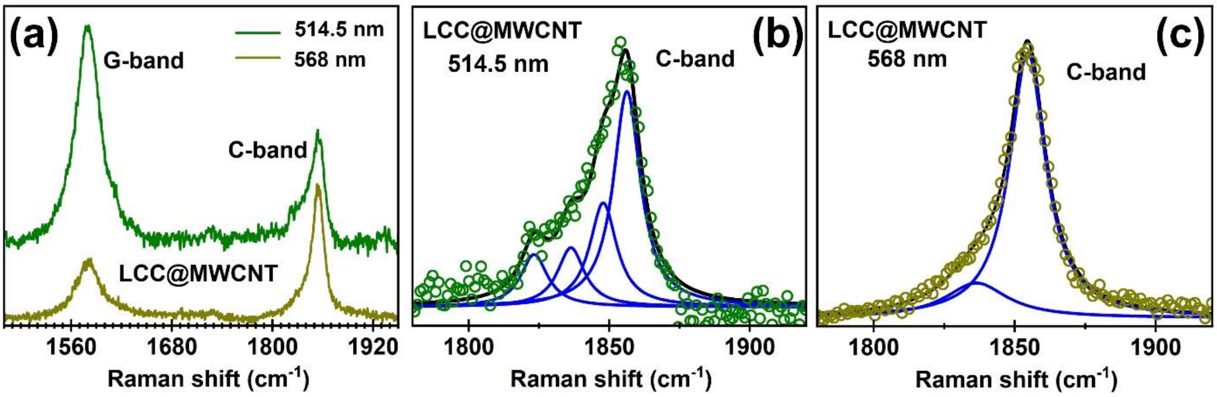

Figure S1: (a), (b) and (c) Representative Raman spectra obtained at 293 K using 514.5 nm and 568 nm lasers. In **(a),** the G-band from the host CNT is around 1580 cm^{-1} and the C-band is around 1850 cm^{-1}. **(b)** using 514.5 nm and **(c)** using 568 nm, show the C-band, where the open symbols are the experimental data, the black solid curve is the fitting result using Lorentzian curves (navy blue solid curves). LCC@MWCNT stands for LCC encapsulated by MWCNT.

Plots of the thermodynamic observable for the full set of LCC:

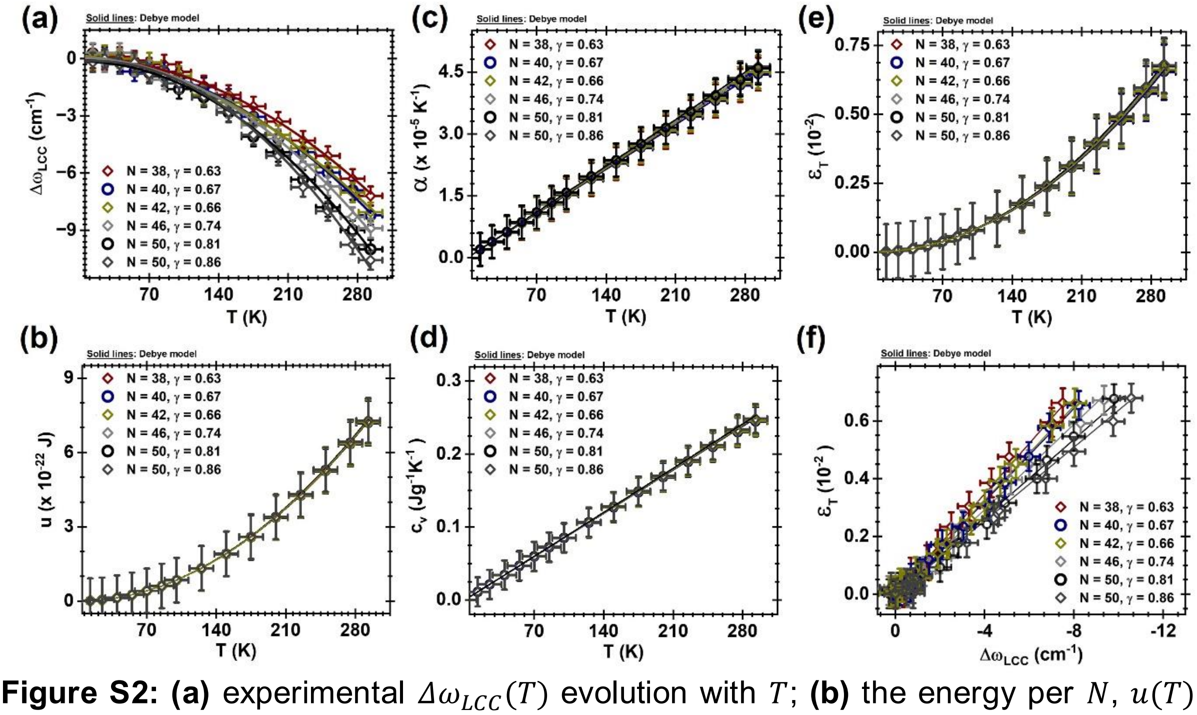

Figure S2: (a) experimental $\Delta\omega_{LCC}(T)$ evolution with T; **(b)** the energy per N, $u(T)$, presents a quadratic, universal, and unified behavior with T; **(c)** $\alpha(T)$ shows a linear universal behavior with T; **(d)** the heat capacity per N, $c_v(T)$, presents a linear, universal and unified behavior with T; **(e)** A T^2 universal dependence is observed for the thermal strain $\varepsilon_T(T)$; **(f)** Every LCC presents a distinct linear dependence of ε_T with $\Delta\omega_{LCC}(T)$.

The Debye model for LCC:

Under the Debye model formalism (from now on DMF), the canonical partition function for LCC is given by **[1]**:

$$lnZ = -N\frac{T}{T_{LCC}}\int_0^{\frac{T_{LCC}}{T}} ln[1-exp(-x)]dx, \qquad \textbf{(S1)}$$

where $x=\frac{\hbar\omega_{ph}}{k_bT}=\frac{T_{ph}}{T}$, ω_{ph} is the phonon frequency and T_{ph} is the phonon equivalent temperature. Upon integration by parts, **Equation S1** becomes:

$$lnZ \approx N\frac{T}{T_{LCC}}\int_0^{\frac{T_{LCC}}{T}} \frac{x}{e^x-1}dx. \qquad \textbf{(S2)}$$

For $T<<T_{LCC}$, $\frac{T_{LCC}}{T}\rightarrow\infty$ and **Equation S2** is simplified to:

$$lnZ \approx N\frac{T}{T_{LCC}}\int_0^{\infty} \frac{x}{e^x-1}dx = N\frac{T}{T_{LCC}}\left(\frac{\pi^2}{6}\right) = \frac{Nk_b\pi^2}{6\hbar}\frac{T}{\omega_{LCC}(T)}, \qquad \textbf{(S3)}$$

where $\int_0^{\infty}\frac{x}{e^x-1}dx = \frac{\pi^2}{6}$. As thoroughly described by Costa and collaborators **[1]**, the LCC's internal energy per N,$u(T)$, $c_v(T)$ and $\alpha(T)$ must contain one term representing the Debye approximation plus corrections involving derivatives of $\omega_{LCC}(T)$. This way $u(T)$ is:

$$u(T) = \frac{k_bT^2}{N}\frac{d(lnZ)}{dT} = \frac{k_b^2\pi^2}{6\hbar}\frac{T^2}{\omega_{LCC}(T)}\left[1-\frac{T}{\omega_{LCC}(T)}\frac{d\omega_{LCC}}{dT}\right], \qquad \textbf{(S4)}$$

Equation S4 leads to the heat capacity per N - $c_v(T)$:

$$c_v(T) = \frac{du}{dT} = \frac{(k_b\pi)^2}{3\hbar}\frac{T}{\omega_{LCC}(T)}\left\{1-2\frac{T}{\omega_{LCC}(T)}\frac{d\omega_{LCC}}{dT}+\frac{T^2}{\omega_{LCC}^2(T)}\left[\left(\frac{d\omega_{LCC}}{dT}\right)^2-\frac{\omega_{LCC}}{2}\frac{d^2\omega_{LCC}}{dT^2}\right]\right\}, \qquad \textbf{(S5)}$$

being $\alpha(T)$ given by:

$$\alpha(T) = \frac{-\delta}{2\Theta^2}\frac{c_v(T)}{a_{C-C}} = \frac{42(k_b\pi)^2}{3\hbar m a_{C-C}^2}\frac{T}{\omega_{LCC}^3(T)}\left\{1-2\frac{T}{\omega_{LCC}(T)}\frac{d\omega_{LCC}}{dT}+\frac{T^2}{\omega_{LCC}^2(T)}\left[\left(\frac{d\omega_{LCC}}{dT}\right)^2-\frac{\omega_{LCC}}{2}\frac{d^2\omega_{LCC}}{dT^2}\right]\right\}, \textbf{(S6)}$$

where a_{C-C}=1.37 Å is the average C-C distance, $\Theta=\frac{m[\omega_{LCC}(T)]^2}{2}$, $\delta=\frac{-21m[\omega_{LCC}(T)]^2}{a_{C-C}}$ and $\varepsilon = \frac{ma_{C-C}^2\left(\omega_{LCC}^0\right)^2}{72}$.

References:

[1] N. L. Costa et al., Thermodynamics of Linear Carbon Chains, Phys. Rev. Lett. 126, 125901 (2021).
